# Supplementary material for: Selective Laser Trabeculoplasty in the Treatment of Ocular Hypertension and Open-Angle Glaucoma: Clinical Review
Source: J Clin Med. 2021 Jul 27;10(15):3307. doi: 10.3390/jcm10153307 (PMC8347751; doi:10.3390/jcm10153307)
Supplement: Supplementary file 1 [file jcm-10-03307-s001.zip › jcm-1277395-supplementary.pdf]

Supplementary materials

Search strategy overview

PubMed

(((selective[All Fields] AND ("lasers"[MeSH Terms] OR "lasers"[All Fields] OR "laser"[All Fields]) AND ("trabeculectomy"[MeSH Terms] OR "trabeculectomy"[All Fields] OR "trabeculoplasty"[All Fields])) OR "slt"[All Fields]) OR (("lasers"[MeSH Terms] OR "lasers"[All Fields] OR "laser"[All Fields]) AND ("trabeculectomy"[MeSH Terms] OR "trabeculectomy"[All Fields] OR "trabeculoplasty"[All Fields]))) AND (((((("glaucoma, open-angle"[MeSH Terms] OR ("glaucoma"[All Fields] AND "open-angle"[All Fields]) OR "open-angle glaucoma"[All Fields] OR ("open"[All Fields] AND "angle"[All Fields] AND "glaucoma"[All Fields]) OR "open angle glaucoma"[All Fields]) OR ("glaucoma, open-angle"[MeSH Terms] OR ("glaucoma"[All Fields] AND "open-angle"[All Fields]) OR "open-angle glaucoma"[All Fields] OR ("open"[All Fields] AND "angle"[All Fields] AND "glaucoma"[All Fields]) OR "open angle glaucoma"[All Fields])) OR ("ocular hypertension"[MeSH Terms] OR ("ocular"[All Fields] AND "hypertension"[All Fields]) OR "ocular hypertension"[All Fields])) OR OAG[All Fields]) OR OHT[All Fields]) OR (exfoliative[All Fields] AND ("glaucoma"[MeSH Terms] OR "glaucoma"[All Fields]))) OR XFG[All Fields])

Number of results: 3 139

When restricted to the last 5 years 1 465

Abstracts: 1 001

Full articles: 109

Additionally we concluded the possible source to be from previous systematic reviews and meta-analysis studies we found 656 studies for this criterion

Embase (Elsevier)

'selective laser trabeculoplasty'/exp OR 'selective laser trabeculoplasty' OR slt OR 'laser trabeculoplasty'

AND 'open angle glaucoma'/exp OR 'open angle glaucoma' OR 'intraocular hypertension' OR pseudoexfoliation

Number of results: 1 066

Abstracts: 596

Full articles: 98

Cochrane CENTRAL

ID Search

#1 ("selective laser trabeculoplasty"):ti,ab,kw OR (laser trabeculoplasty):ti,ab,kw OR (SLT):ti,ab,kw (Word variations have been searched)

#2 (open angle glaucoma):ti,ab,kw OR (OAG):ti,ab,kw OR ("open angle glaucoma"):ti,ab,kw OR ("ocular hypertension"):ti,ab,kw OR (exfoliative glaucoma):ti,ab,kw (Word variations have been searched)

Number of results: 674 and 4 822 respectively for #1st and #2nd search modalities

Supplementary materials

Abstracts: 1 012

Full articles: 127

Web of Science

TOPIC: (selective laser trabeculoplasty) OR TOPIC: (laser trabeculoplasty) OR TOPIC: (SLT) OR TOPIC: (open-angle glaucoma) OR TOPIC: (OAG) OR TOPIC: (ocular hypertension) OR TOPIC: (OH) OR TOPIC: (exfoliative glaucoma) OR TOPIC: (XFG)

Refined by: DOCUMENT TYPES: (ARTICLE)

Timespan: Last 5 years. Indexes: SCI-EXPANDED, SSCI, A&HCI, CPCI-S, CPCI-SSH, BKCI-S, BKCI-SSH, ESCI, CCR-EXPANDED, IC.

Number of results: 64 784

Abstracts: 2 014

Full articles: 123
